# The Impact of Sex on Arterial Ischemic Stroke in Young Patients: From Stroke Occurrence to Poststroke Consequences

**DOI:** 10.3390/children8030238

**Published:** 2021-03-18

**Authors:** Beata Sarecka-Hujar, Ilona Kopyta

**Affiliations:** 1 Department of Basic Biomedical Science, Faculty of Pharmaceutical Sciences in Sosnowiec, Medical University of Silesia in Katowice, Kasztanowa Str 3, 41-200 Sosnowiec, Poland; 2 Department of Pediatric Neurology, Faculty of Medical Sciences in Katowice, Medical University of Silesia in Katowice, Medykow Str 16, 40-752 Katowice, Poland; ilonakopyta@autograf.pl

**Keywords:** arterial ischemic stroke, stroke, children, young adults, sex, gender, clinical presentation, poststroke outcomes

## Abstract

The male sex has been suggested to predominate in paediatric patients with arterial ischemic stroke (AIS), especially in newborns. The explanation for this phenomenon remains unsatisfactory since it focuses on the analysis of the potential relationship with trauma and arterial dissection. In turn, in some populations of young adults, men suffer from AIS more frequently than women, which may be related to the protective role of oestrogen. On the other hand, certain data indicate that women dominate over men. Some of the disparities in the frequencies of particular symptoms of AIS and poststroke consequences in both children and young adults have been suggested; however, data are scarce. Unfortunately, the low number of studies on the subject does not allow certain conclusions to be drawn. For adults, more data are available for patients aged over 60 years, the results of which are more obvious. The present literature review aimed to discuss available data on the prevalence of AIS, its clinical presentations, and poststroke consequences in regard to the sex of young patients. We considered young patients to be children from birth up to the age of 19 years of life and young adults to be individuals up to the age of 55 years. The role of sex hormones in AIS and possible gender differences in genetic risk factors for AIS were also discussed briefly.

## 1. Introduction

Arterial ischemic stroke (AIS) in young patients, from the neonatal period to young adulthood, has a multifactorial determination. It carries detrimental consequences to anyone affected by the disease (e.g., hemiparesis, intellectual delay, recurrence of the disease, and poststroke epilepsy) [1]. However, risk factors for stroke in children and young adults differ from each other.

Maternal factors, such as chronic diseases, including metabolic and endocrine disorders, as well as genetic and acquired coagulation disorders, and environmental ones, especially stimulants, are crucial in the stroke pathology during the neonatal period. Subsequently, factors related to the delivery itself, such as infection or arterial hypertension, and finally congenital infections and neonatal infections, coagulation disorders in the newborn, and vascular defects are of great importance. The neonatal period is the time with the highest incidence of ischemic stroke in the whole infant population. Paediatric AIS, defined as occurring in the period from 29 days of age to 18 years of age, is also characterized by a multifactorial conditioning. The most important risk factors for AIS are arteriopathies, including focal arteriopathy of childhood (FCA), followed by congenital and acquired heart diseases, prothrombotic states, injuries, and intoxications [2]. In young adults, for whom the upper age limit is set at 40–55 years by different authors, the most important risk factors are hypertension, metabolic diseases, and coagulation disorders [3]. Apart from age-specific factors, the patient’s sex plays an important role in each of the identified age groups; it is important not only for the clinical presentation in the acute phase of stroke and its type, but also for the prognosis and consequences of a history of cerebral ischemia.

Some of the data show a relationship between sex and AIS, while others do not confirm such finding. AIS has been demonstrated to occur more often in males in the paediatric population [4,5,6,7], whereas in the population of older adults, women more often suffer from AIS, also with a worse long-term prognosis [8]. Different risk factors for stroke in young women and men can also be distinguished. Abanoz et al. [9] reported migraine with aura as an independent risk factor for AIS in women but not in men.

The causes of male predominance in paediatric patients are poorly understood. However, more frequent infections and head injuries in male children have been reported as the possible factors related to this phenomenon. In turn, in the adult population, the more frequent occurrence of stroke itself, the type of stroke, and its consequences in one of the sexes are considered in the context of hormonal determinants, which is also closely related to the age of patients. This context in the developmental population is much less explored and explained.

The present literature review examines data on the prevalence of AIS, its clinical presentations, and poststroke consequences in regard to the sex of young patients. Paediatric patients from birth to 19 years of life and young adults aged below 55 years are considered young cases. The role of sex hormones in AIS and possible gender differences in genetic risk factors for AIS are also discussed briefly.

## 2. Methods

We searched PubMed, Scopus, Google Scholar, and Embase using combinations of the following keywords: “arterial ischemic stroke,” “arterial ischemic stroke,” “stroke,” “sex,” “gender,” “children,” “paediatric,” “young,” and “young adults” (last search in January 2021).

Available data define “young adults” with different upper limits of age. Most frequently, an adult person aged below 45 years is considered “young adult”; however, there are numerous papers analysing adult patients aged below 55 years or even 60 years as “young adults.”

In the present literature review, we included results analysing patients suffering from arterial ischemic stroke. For some data on AIS, it was discussed together with transient ischemic attacks (TIA); thus the articles on TIA were also included to the review. In addition, studies on both perinatal stroke, defined as cerebral circulatory disturbance between the 20th week in prenatal life and the 28th day of life, and neonatal stroke, occurring till the 28th day of life [10,11], were discussed. On the other hand, we excluded from the discussion data on haemorrhagic stroke and cerebral sinovenous thrombosis (CSVT), although some of the studies analysed cases with AIS and haemorrhagic stroke jointly.

We followed the AIS criteria published by Golomb et al. [11]: “(1) neurological deficit of acute onset, or seizure alone in neonates; and (2) radiographic image(s) [magnetic resonance imaging (MRI) and computer tomography (CT)] showing cerebral parenchymal infarct(s) conforming to known arterial territory(ies) and corresponding to clinical manifestations.”

The search process using all the keywords allowed us to identify a total of 8584 full-length articles written in English. Next, after excluding papers related to patients aged over 55 years or presenting diseases other than AIS, 262 articles were left. After another screening, duplicates and items not relevant were removed, leaving us with 93 articles that we found the most pertinent on the topic.

## 3. Sex Distribution in Different Populations of Young Patients with AIS

### 3.1. Paediatric Population

AIS is among the 10 most common causes of death in children [12,13,14]. The incidence of stroke in the paediatric population is estimated at about 2–3 new cases per 100,000 paediatric population per year [11,15,16]. The results of a 16-year population-based study by the Canadian Pediatric Ischemic Stroke Registry enabled us to recruit 1129 children with stroke. Based on such sizeable group of patients, the frequency of childhood stroke was estimated at 1.72 per 100,000 per year, and the frequency of neonatal stroke at 10.2 per 100,000 live births [17].

Differences in the incidence of stroke reported in the literature data may result from the differences in the ages of the patients enrolled in the study. Usually, neonatal stroke is treated as a problem separate from childhood stroke. On the other hand, the upper age limit for paediatric patients may be set from 16 to 20 years of age. Overall, in the paediatric population, stroke occurs more frequently in the male sex [5]. One of the explanations may be slightly higher birth rates for males compared with females in some countries with a proportion of 1.05:1, respectively (i.e., 51.2% boys) [11,18,19,20].

Table 1 demonstrates the prevalence of the female/male sex in children with AIS from selected studies performed in different populations. The highest percentages of boys suffering from AIS were reported by Coen Herak [6] and Lopez-Espejo et al. [7] (i.e., 66% and 62.5%, respectively). Similarly, a study by Sultan et al. [5], which was performed in collaboration with several medical centres within the International Pediatric Stroke Study (IPSS), demonstrated almost 59% boys in a sizeable group of paediatric patients with AIS.

**Figure 1:**
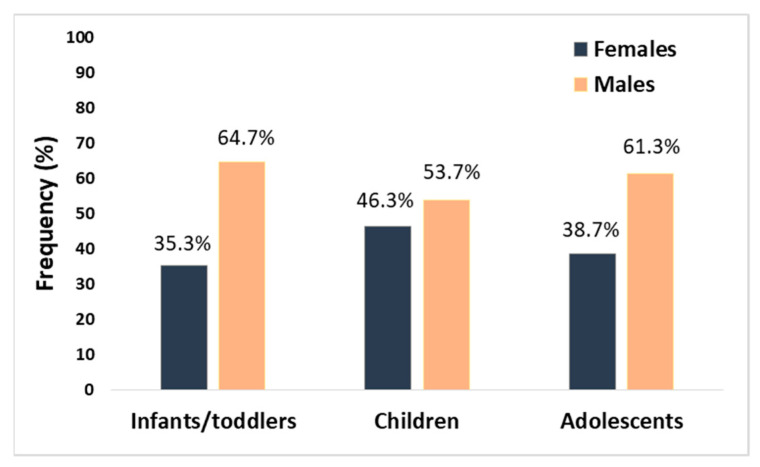
Distribution of sex in the age subgroups of paediatric patients with AIS (the differences did not reach statistical significance; the graph is based on data partially published previously [4]).

In our study, which was based on 89 children with AIS (aged from the 28th day of life to 18 years), the male sex also predominated in the total group (58% of cases were males) as well as in the subgroups according to age [4]. Figure 1 shows the sex prevalence in age subgroups of children with AIS analysed by us earlier (i.e., infants/toddler (aged between the 28th day of life and the 35th month of life), children (aged 3 to 11 years), and adolescents (aged between the 12th year of life and the 18th year of life)). The particular numerical advantage in the incidence of stroke in males was visible in the groups of infants and toddlers and adolescents; in the group of infants, this advantage was almost twofold [4].

An extremely high percentage of males among patients with perinatal stroke was observed by Gelfand et al. [27], who accounted for 77% of all strokes; however, the number of analysed children was very low (*n* = 13).

A British 1-year study determining the incidence of new childhood stroke cases in a population of Southern England of around 6 million children identified 96 children with AIS [14]. The authors did not find any difference in stroke rate between the female and male paediatric patients. The two groups of patients described by Kopyta et al. [4] and Mallick et al. [14] are comparable in terms of the number of recruited patients. There is, however, a difference in the upper age limit of the patients between the studies (18 years old in the Polish study and 16 years old in the British study), and this fact may be important for the obtained results.

Numerous studies on neonatal and childhood stroke conducted in different geographic areas (i.e., Europe, Saudi Arabia, Canada, Israel, and Turkey) also indicated a higher incidence of AIS in male children [12,28,29,30,31,32,33,34,35]. In a study performed in 2004 by Golomb et al. [28] on a group of 66 newborns with AIS and 32 newborns with sinovenous thrombosis of the brain at the gestational age of over 36 weeks, boys dominated in both subgroups. Male newborns with AIS were slightly frequent than girls, but the explanation for the predominance of the male sex remains ambiguous [28]. Additionally, in Saudi Arabian newborns with perinatal stroke consisting of 104 children with the diagnosis from birth up to 12 months of life, the male sex predominance was described [29].

Another study by Golomb et al. [11], which was performed in cooperation with the International Pediatric Stroke Study (IPSS) in 2003–2007, included nearly 1200 children from the neonatal period to 19 years of age with both AIS and sinovenous thrombosis (SVT). Of the total recruited children, 60% were males. The ages of the children with AIS were comparable between sexes. The mean age of the male patients was 6.8 years old, while the mean age of the female patients was 7.4 years old. Most often, the stroke in the group of males was preceded by a head injury (out of 60 children with head trauma preceding stroke, 75% were males); however, the stroke also occurred in 58% of the male children without any head injury [11]. Due to the higher incidence of AIS in the male paediatric patients, the authors suggested a preceding head injury and risky behaviours as a possible explanation for such disproportion. Previously, head or neck trauma (within the past 12 weeks) and minor acute infection (within the past 4 weeks) were observed to be risk factors for AIS in a group of 126 children with stroke (odds ratio (OR) = 9.0, 95% CI: 3.2–25.1, *p* < 0.001, and OR = 3.9, 95% CI: 2.0–7.4, *p* < 0.001, respectively) [36]. On the other hand, assuming that risky behaviours are undertaken with equal frequency by males and females, male paediatric patients may be more prone to traumatic arterial dissection, possibly related to yet undiagnosed chromosome X-related disorders [11]. In the case of infection, Helmuth et al. [37] observed that 10 out of 15 children with AIS and varicella less than 12 months before were male (67%). Similarly, Fullerton et al. [38] demonstrated higher frequency of males among children with AIS and acute infection (8 out of 14 cases, i.e., 57%). The authors observed that 5 patients with parvovirus B19 had a distinct arteriopathy involving a long segment of the intracranial anterior circulation [38].

Authors from California analysed from a medical records database in the period 1991–2000 all data on the discharge of children aged 1 month to 19 years with a diagnosis of stroke [12]. Among more than 2000 cases of ischemic stroke, haemorrhagic stroke, and subarachnoid haemorrhage, male patients were numerically predominant. As in the previously cited publication, the relationship between head injuries and stroke in boys was taken into account; however, even after eliminating this risk factor, the male gender predominated.

A study by Amlie-Lefond et al. [39] performed on 676 children with pediatric AIS (age of stroke onset between 29th day of life and 19 years of life) indicated again the male sex predominance (i.e., 59% of the whole analysed group were boys). On the other hand, a study by Szaflarski et al. [40] showed a change in the percentage of females when groups of perinatal stroke and late stroke (i.e., children aged > 1 month) were analysed. Females slightly predominated among perinatal strokes, while the percentage of female patients decreased in late strokes.

Some of the studies demonstrated equal or almost equal distribution of girl and boys in a group of children with AIS [21,22,26]; however, the studies were performed on a small number of cases (*n* = 86, *n* = 73, and *n* = 24, respectively). On the other hand, data by Hills et al. [36] demonstrated that the male sex cannot be considered a risk factor for AIS in children from the US (OR = 1.2, 95% CI: 0.7–1.9, *p* = 0.56). In turn, in the prospective multicentre study by Nowak-Göttl et al. [25], girls with AIS predominated over boys (53% vs. 47%).

### 3.2. Young Adults

Previously, it was demonstrated that the proportion of all strokes in people under the age of 55 years increased from 12.9% to 18.6% in the US [41]. The increasing trend was especially visible among white patients aged 20–44 years. At the same time, a decreasing trend in AIS occurrence among white people aged 75–84 was demonstrated [41]. Such a tendency in young American patients may result from an increasing number of main risk factors for AIS (i.e., diabetes mellitus and obesity).

In Table 2, the prevalence of the female/male sex in young adults with AIS from selected studies performed in different populations is demonstrated.

A Swedish study reported that the first-ever stroke cases, aged 15–44 years, accounted for 1.4% of the total number of strokes during the period 2001 to 2009 [42]. The authors found that 58% of the young patients with stroke were men. The frequency of male patients with stroke similar to the data by Bergman et al. [42] was demonstrated by Martínez-Sánchez et al. [43] and Spengos and Vemmos [44] (59% and 57%, respectively). The Spanish patients were, however, slightly older (i.e., the patients’ ages ranged from 15 to 50 years) than the patients from Sweden and Greece (Table 2). Both of the studies performed in the south of Europe distinguished the patients aged 15–30 years (so-called “very young group”) from the total group of cases, and surprisingly, the frequency of men differed between the studies (53% in the study by Martinez-Sánchez et al. [43] and 44% in the Greek study [44]).

The highest percentage of men was demonstrated in a retrospective study from South Korea and accounted for 75% [45]; however, the number of analysed patients was lower compared with those in other analysed studies (Table 2). In turn, 69% of Chinese young adults with stroke were men [46]. A comparable percentage of male cases with AIS, 62% each, was demonstrated in studies from Germany and Finland [47,48].

On the other hand, a study from the Netherlands published in 2016 demonstrated that women predominated among stroke patients [49]. The female/male ratio in this country was subsequently confirmed in another study by Ekker et al. [50], which included a sizeable group of patients. The two studies differed, however, in terms of study design. A study analysing among others over 5000 index strokes in patients younger than 55 years showed that in two age subgroups (i.e., 25–34 and 35–44 years old), more women had strokes than men (incidence rate ratio: men/women values of 0.70 and 0.87, respectively) [51]. On the contrary, in a subgroup aged between 45–54 years, more strokes among men were demonstrated.

Some studies indicated no special predominance of one of the sexes [52,53]. The studies, however, included a very low number of patients (*n* = 54 and *n* = 131, respectively), especially in the Nigerian study [52]. One of the studies, performed on Polish patients, was conducted on a group of children aged 1 year up to young adults aged 50 years [54], which may affect the obtained results (i.e., lack of differences in frequency between men and women).

## 4. Clinical Presentation of AIS According to Patients’ Sex

### 4.1. Symptoms of AIS in Paediatric Population

The clinical symptoms of neonatal stroke are nonspecific and are most often characterized by disturbances in consciousness and seizures. In a group of children described by deVeber et al. [17], seizures were observed in 88% of the neonates aged below 28 days and born at ≥36 weeks of gestation, making the symptom the most common in neonatal stroke. Consecutively, disturbances of consciousness and disorders related to the nature of respiratory and circulatory failure were observed in this group of patients [17]. Probably due to the unequivocal high frequency of the described nonspecific symptoms observed in the entire group of infants with ischemic stroke, no division by sex was made.

On the contrary, in a nationwide population-based study on paediatric ischemic stroke, a division in the neonatal group was done [55]. The study covered the entire child population (i.e., from birth to 18 years of age) from Denmark, and it lasted 12 years. During this period, neonatal stroke was diagnosed in 51 children, 31 of whom were males. Similar to the study by deVeber et al. [17], seizures were the most common symptoms of AIS, observed in about 85% of the patients [55].

In our previous study, a significant male predominance was found in total anterior circulation infarct (TACI) stroke with a 2:1 male/female ratio. In the posterior circulation infarct (POCI) stroke type, the ratio was 10:1 [4]. A slight predominance of female patients was seen in the lacunar anterior circulation infarct (LACI) subgroup. In addition, the prevalence of FCA significantly differed between stroke subtypes among female paediatric patients (*p* = 0.046) [4].

Clinical symptoms of the acute phase of stroke depend on the location of the vascular pathology and the effect of the swelling of the brain tissue surrounding the ischemic focus. In addition, in the youngest children, acute brain damage is associated with the occurrence of nonspecific clinical symptoms, such as consciousness disturbances, seizures, and respiratory failure.

In our previous study, hemiparesis, central paresis of the facial nerve, consciousness disturbances, and aphasia were the most dominant symptoms. No statistical sex-dependent differences in the prevalence of clinical symptoms were observed; however, central type facial nerve palsy and other symptoms significantly differentiated AIS subtypes in boys [4]. In turn, in our patients with POCI stroke, a more frequent occurrence of headaches, dizziness, and consciousness disturbances was demonstrated [4].

A Canadian retrospective study on paediatric stroke presented that 23 out of 158 AIS children had POCI stroke, of whom 21 were over the age of 28 days of life, and among them, 11 were male [56]. Consciousness disorders in the acute stage of the disease were reported in 71% of the analysed group, while almost 40% of the children presented other nonspecific symptoms [56].

The diagnosis of stroke in childhood is difficult and often postponed, which excludes patients who could potentially be treated with intravenous thrombolysis. Many factors can delay the diagnosis of stroke in childhood, including the awareness of the parents of children with, for example, heart defects that the risk of AIS exists, but most of all, the primary care physician. In addition, the performance of a CT scan for early and minor stroke lesions is often a factor in overlooking them. MRI should be the method of choice for suspected AIS in a child.

### 4.2. Symptoms of AIS in Young Adults

Symptoms of AIS also differ between sexes; however, data on the topic are scarce. Data indicate that women experience symptoms like nausea/vomiting, headache, dizziness, and cognitive dysfunction more often than men [57]. Older women were observed to experience nontraditional stroke symptoms, such as pain, change in the level of consciousness–disorientation, and unclassifiable neurologic and nonspecific symptoms, more commonly than older men (28% vs. 19%, respectively; OR = 1.62) [58]. The differences in stroke symptoms between sexes may be the reasons for delays and difficulties in timely diagnosis. A prospective hospital-based study conducted in Qatar, performed in 217 patients with ischemic stroke (mean age of 57.2 ± 13.3 years), demonstrated significant differences between sexes in presenting symptoms of AIS [59]. Dysarthria, swallowing deficits, and gait imbalance were significantly more common in men. Headache as a stroke symptom was especially high in women (68% vs. 31% in men) [59]. Limb paralysis/weakness, convulsions, and coma concerned both sexes equally. Similar to paediatric stroke, posterior circulation stroke was found to be significantly frequent in male patients (24.8% vs. 1.6% in women), whereas partial anterior circulation stroke syndrome was more common in female patients (35% vs. 20.4% in men) [59]. Similarly, a multicentre prospective study across Europe on patients aged 18–55 years revealed men to have posterior circulation stroke more frequently [60]. In a study by Khan and Ibrahim [59], the prevalence of other stroke subtypes (i.e., total anterior circulation stroke syndrome and lacunar stroke syndrome) did not differ between sexes. Interestingly, the authors also observed a significant difference between sexes in regard to time at hospital admission. A higher percentage of women than men were admitted within 3 hours from stroke onset (26.6% vs. 14%, respectively, *p* = 0.04) [59].

A study by Kuruvilla et al. [61] demonstrated that misdiagnosis at the emergency department concerned men and women with AIS with the same frequency, whereas in a group of correctly diagnosed cases, 38.7% were men.

As for the aetiology of stroke, a Chinese study based on 411 young patients with first-ever stroke demonstrated that large-artery atherosclerosis and small-vessel diseases were more prevalent in men, whereas women were affected significantly more commonly by stroke of other determined aetiology [46].

## 5. Poststroke Outcome According to Sex

### 5.1. Poststroke Outcome in Paediatric Population

The outcome of AIS in children depends mainly on the time of the onset of a stroke and on the location or size of the ischemic focus. An unfavourable AIS course and death as a consequence of stroke may occur in the acute phase of the stroke, even during the child’s hospitalization or during follow-up.

Golomb et al. [11] observed no gender differences in case fatality or deficits at discharge. The authors observed that a similar percentage of girls and boys (35% and 32%, respectively) were normal at discharge.

Data from Polish children with AIS demonstrated that the frequencies of particular poststroke deficits did not differ between males and females [4]. However, it was observed that seizures after stroke appeared significantly more commonly in female patients with TACI and POCI stroke, compared with LACI and partial anterior circulation infarct (PACI) stroke (OR = 8.62, *p* = 0.017). In male patients, poststroke hemiparesis also differentiated stroke subtypes; males with TACI and PACI stroke had a higher risk of having hemiparesis compared with males with LACI and POCI stroke (OR = 5.36, *p* = 0.008) [4]. The literature indicates that the frequency of both poststroke seizures and epilepsy is higher in children than in young adults [62,63,64,65]. An especially high percentage of children suffering from seizures after stroke was reported in studies from the Netherlands, Korea, and Brazil and accounted for 32.3%, 26%, and 64.6%, respectively [63,66,67]. In our earlier study, however, male and female paediatric patients experienced early seizures and late remote seizures after stroke with the same frequency [68].

Previously, Westmacott et al. [69] pointed out that male paediatric patients with unilateral neonatal stroke are at an increased risk for long-term cognitive deficits. Boys performed significantly worse in measures of general intellectual capacity, nonverbal reasoning, and processing speed than girls. The authors suggested that the immaturity of the male brain at birth compared with the female one, with its particular susceptibility to damage caused by stroke, may be the explanation for this difference [69]. In our previous study [4], posterior stroke was especially common among males. Injuries of posterior areas, the cerebellum in particular, may have an impact on language processing; thus posterior strokes may cause cognitive deficits [70].

Another poststroke outcome that is of great importance is the recurrence of AIS. A multicentre study in the Vascular Effects of Infection in Pediatric Stroke (VIPS) project enrolled 355 children with AIS and followed them for recurrence [71]. The authors observed that 62.5% of the children with recurrent stroke were males compared with 55.2% of the males in a group without recurrence (hazard ratio (HR) = 1.3, 95% CI: 0.7–2.4, *p* = 0.46), and highest frequency of recurrence concerned children aged 4 to 7 years [71]. In a study by Uohara et al. [72] performed on 107 children with AIS, 11 patients suffered from stroke recurrence, of whom 10 (i.e., 91%) were male. The authors observed a quite high rate of arterial dissection in the paediatric patients with AIS recurrence—five out of nine recurrences of posterior stroke were in patients with dissections (in two of them, thrombophilia coexisted), and all were male [72]. Most recently, extracranial dissection was also associated with the male sex (OR = 3.17, 95% CI: 1.6–6.4, *p* = 0.001) [73].

### 5.2. Poststroke Outcome in Young Adults

In Chinese young adults with AIS aged below 50 years, young women had poorer outcomes defined as having a modified Rankin Scale score of 3–6 at 12 months. Except for the female sex, the National Institutes of Health Stroke Scale (NIHSS) score and pneumonia were reported as independent factors for poor outcome [74]. The authors also observed systolic blood pressure at admission to be significantly lower in women than in men [74].

In a group of 606 ischemic stroke patients aged 18 to 50 years, of whom 20% died, a cumulative 20-year mortality among 30-day survivors was higher in men than in women [75]. Some tendencies towards a relationship between female gender, posterior circulation territory, and poor outcome after stroke were observed in younger adults with first-ever stroke studied in Israeli [76].

A study by Roivainen et al. [64] observed that there were no differences in sex distribution among patients who experienced acute symptomatic seizures after stroke; however, in a group of seizure-free patients, men predominated over women (63% vs. 37%). Similarly, a study on almost 700 patients aged 18–50 years reported also no sex differences in poststroke epilepsy, and the percentage of men was slightly lower than that of women (44.3% vs. 55.7%) [77]. Additionally, a study by Naess et al. [78] confirmed no relationship between sex and the risk of developing poststroke seizures in patients aged 15–49 years. Sex also did not influence functional outcome after stroke (*p* = 0.30) [78]. Other studies demonstrated no sex relationship with poststroke depression in young patients [79] or with poststroke delirium [80].

**Figure 2:**
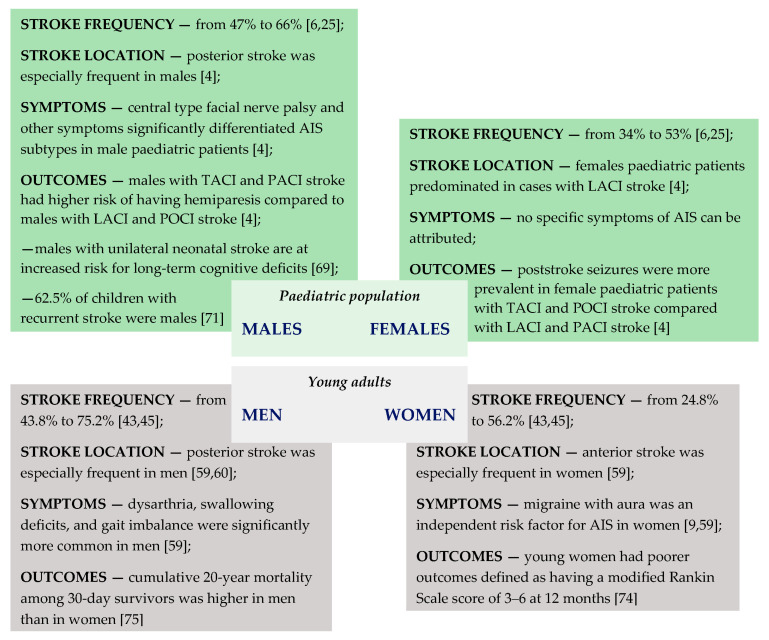
Summary of the literature review on the frequency of AIS occurrence [6,25,43,44], stroke location [4,59,60], stroke symptoms [4,9,59], and poststroke outcomes [4,69,71,74,75] according to sex in both paediatric patients and young adults; AIS—arterial ischemic stroke; LACI—lacunar anterior circulation infarct; TACI—total anterior circulation infarct; PACI—partial anterior circulation infarct; POCI—posterior circulation infarct.

Figure 2 summarizes data on the frequency of AIS occurrence, location, symptoms, and poststroke outcomes according to sex in both paediatric patients and young adults. 

## 6. The Role of Hormones in AIS

Since in the adult premenopausal age group majority of stroke patients are male and there is a rapid change to the detriment of women afterwards, female hormones have become a major focus of investigation. Oestrogen and oestradiol have been found to have a neuroprotective effect (in both in vivo and in vitro studies). Over 2 decades ago, Simpkins et al. [81] reported that oestrogens may be promising in protecting neurons against the neurodegenerative effects of stroke. Female rats were ovariectomized, and then various oestrogen preparations before or after middle cerebral artery (MCA) occlusion were administered to them. The authors demonstrated a decrease in mortality as well as ischemic area [81]. Another study performed on Wistar rats showed that 17β-oestradiol (E2) is neuroprotective in male rats when given at the acute stage of the ischemia, and bazedoxifene (BZA), which is a third generation of selective oestrogen receptor modulators (SERM) at clinically relevant plasma levels, mimics the neuroprotective action of E2 and could be, therefore, a candidate for stroke treatment [82].

However, as the stroke incidence peak among women is much later (around 80s) than the loss of ovary function (around 50s), it is clear that there are other factors contributing to the result. What is more, results of clinical trials with the use of oestradiol among the population of postmenopausal women were discouraging—the mechanism is yet unclear, but a higher incidence of strokes and fatal outcomes was observed [83,84,85,86]. The neuroprotective effect of progesterone treatment alone or in combination with oestrogen was also postulated [86].

This hypothesis fails in the paediatric population if we consider that in a group of preadolescents (0–12 years old) and adolescents (13–19 years old), there was still a numerical predominance of boys [11]. However, an increase of AIS risk associated with elevated testosterone levels among boys was described [87]. For each 1 nmol/L increase in testosterone in boys, the authors calculated that the odds of cerebral thromboembolism were increased 1.3-fold [87].

## 7. Genetic Risk Factors for AIS According to Sex

Several studies are available showing that genetic risk factors for AIS may be sex dependent; however, data on the topic are scarce. AIS is a multifactorial disease, and genetic risk factors may have a higher impact on stroke pathology in younger patients than in elderly. Our previous study on children with AIS demonstrated that both carriers of the T allele of 677C>T polymorphism in the *MTHFR* gene and the T allele itself are associated with an increased risk of AIS in male paediatric patients suffering from stroke (odds ratio (OR) = 3.09, *p* = 0.023, and OR = 3.09, *p* = 0.009, respectively) [88]. In addition, the T allele was transmitted significantly more often from heterozygous parents to male cases. Similarly, preliminary data by Coen Herak et al. [89] reported a significant gender-specific difference of *FXIII-A* Val34Leu polymorphism in males with childhood AIS compared with healthy males (OR = 2.44, 95% CI: 1.07–5.53). Such relationship was not observed in male patients with perinatal AIS and in females with childhood or perinatal AIS.

On the other hand, another study performed in the same group of patients analysing the correlation between 98G > T polymorphism in the E-selectin gene and AIS did not reveal such associations in sex subgroups [90].

In a study on a population of 657 adult patients with AIS performed by Gromadzka et al. [91], a correlation between gender and *APOE* polymorphism in predicting poststroke outcome was demonstrated. The E4 genotype of the *APOE* gene was found to be a significant independent positive predictor of death within 1 year after AIS in men [91].

On the other hand, in the case of -455G>A polymorphism in the fibrinogen beta gene, an association between the carrier state of the A allele and worse survival in women suffering from AIS who simultaneously smoked was demonstrated compared with nonsmoking women with the GG genotype [92].

A case study described two young men with a rare *FV* Leiden mutation [93]. One of the men during a recurrence of ischemia presented a sudden onset of right hemiplegia with right facial paralysis. The patient was treated with intravenous recombinant tissular plasminogen activator 2.5 h from the onset of symptoms and showed signs of clinical improvement at the end of 2 h. The second patient had a quantitative deficiency of plasminogen, and he was a heterozygote for *FII* 20210A mutation. He presented ischemic sequelae in the vicinity of the posterior cerebral artery [93].

## 8. Conclusions

Data from numerous studies indicate a higher incidence of ischemic stroke in male paediatric patients from the neonatal period to adolescence. The explanation for this fact remains unsatisfactory and focuses on the potential relationship with trauma and arterial dissection. In young adults, men also predominate in cases with AIS, which may be due to the protective role of oestrogen in women. In turn, in elderly, women most often predominate among stroke patients, which may result from decreased oestrogen neuroprotection. Data also indicate disparities in the frequencies of particular symptoms of AIS and poststroke consequences in both children and young adults. In addition, some differences between sexes were reported in young patients in regard to genetic risk factors for AIS. Unfortunately, the amount of data on the topic is not sufficient and does not allow certain conclusions to be drawn. On the other hand, for patients over 60 years of age, more data are available, with more obvious results.

Despite all these doubts related to the importance of young patients’ gender in the acute phase of AIS and poststroke consequences, such analyses may become a contribution to in-depth research in this field. The explanation of the significance of the sex in childhood stroke seems to be more difficult compared with that in adult stroke patients since the importance of gender hormones should be excluded, at least in the preadolescent population. However, knowledge on stroke symptoms specific to one of the sexes may have an impact on reducing the number of misdiagnoses or diagnosis delays, which in turn will affect the length of hospitalisation and degree of poststroke outcomes. Earlier identification of risk factors for AIS specific to females and males may help to avoid at least some incidents of acute cerebral ischemia.

## Figures and Tables

**Table 1:** Prevalence of females/males in selected studies performed in different populations of children with arterial ischemic stroke (AIS).

Study	Type of the Study	Population	Number of Cases	Age at Time of AIS	Females *n* (%)	Males *n* (%)
Kopyta et al. [4]	Retrospective	Patients hospitalized in the Department of Pediatric Neurology of the Medical University of Silesia in Katowice (Poland)	89	From 1 month to 18 years old	37 (42)	52 (58)
Sultan et al. [5]	Cross-sectional analysis	Patients from the International Pediatric Stroke Study (IPSS) registry	1652	From 28 days of age to 18 years old	679 (41.1)	973 (58.9)
Coen Herak et al. [6]	Retrospective	Patients admitted to the Department of Neuropediatrics, Children’s Hospital Zagreb, and the Department of Pediatric Neurology, University Hospital Centre Zagreb (Croatia)	73	Below 18 years old	25 (34)	48 (66)
Lopez-Espejo et al. [7]	Single centre-prospective observational study	Patients admitted to the Pontifical Catholic University of Chile’s Clinical Hospital (Chile)	119	Between 30 days and 18 years old	45 (37.8)	74 (62.2)
deVeber et al. [17]	Prospective, national population-based study	Patients registered in the Canadian Pediatric Ischemic Stroke Registry diagnosed with AIS in any of the 16 Canadian acute care paediatric hospitals (Canada)	933	From birth to 28 days and from 29 days to 18 years	420 (45)	513 (55)
Bohmer et al. [21]	Retrospective	Patients admitted to the Department of Pediatrics, University Hospital of Muenster (Germany)	86	>29 days and <18 years old	42 (49)	44 (51)
Steinlin et al. [22]	Retrospective	Patient cohorts with FCA from Switzerland and Australia	73	From 1 month to 18 years old	36 (49.3)	37 (50.7)
Per et al. [23]	Retrospective	Patients diagnosed at Erciyes University Children’s Hospital in Central Anatolia (Turkey)	130	From 1 month to 16 years old	62 (47.7)	68 (52.3)
Rambaud et al. [24]	Retrospective cohort study	Patients with a first-ever ischemic stroke hospitalized in 10 French academic centres (France)	60	10–18 years old	28 (47)	32 (53)
Nowak-Göttl et al. [25]	Prospective multicentre study	Patients recruited from different geographic areas of Germany	198	from 6 months to 16 years old	105 (53)	93 (47)
Masri and Al-Ammouri [26]	Retrospective	Patients from the Child Neurology Clinic at Jordan University Hospital (Jordan)	24	1 month to 13 years (median: 5 years)	12 (50)	12 (50)
		Total, *n*	3437		1491	1946

FCA—focal cerebral arteriopathy of childhood.

**Table 2:** Prevalence of females/males from selected studies performed in different populations of young adults with AIS.

Study	Type of the Study	Population	Number of Cases	Age at Time of AIS	Females *n* (%)	Males *n* (%)
Bergman et al. [42]	Retrospective, nationwide cohort study	Cohort was based on three registries: the Swedish Stroke Register, the Swedish National Patient Register and the National Board of Health and Welfare, and the Population Register (Sweden)	2599	15–44	1088 (41.9)	1511 (58.1)
Martínez-Sánchez et al. [43]	Retrospective	Patients admitted at the Stroke Center, Department of Neurology, Hospital Universitario La Paz, Madrid (Spain)	310/38 *	15–50/ 15–30 *	128 (42.3)/ 18 (47.4) *	182 (58.7)/ 20 (52.6) *
Spengos and Vemmos [44]	Prospective, cooperative observational study	Patients admitted to the Departments of Neurology and Clinical Therapeutics of the Athens University Medical School (Greece)	253/48 *	15–45/ 15–30 *	110 (43.5)/ 27 (56.2) *	143 (56.5)/ 21 (43.8) *
Kwon et al. [45]	Retrospective	Patients admitted to the Department of Neurology, University of Ulsan, Asan Medical Center, Seoul (South Korea)	149	15–44	37 (24.8)	112 (75.2)
Tang et al. [46]	Retrospective cohort study	Young adults with ischemic stroke consecutively admitted to the Peking Union Medical College Hospital (China)	411	18–50	129 (31.4)	282 (68.6)
Aigner et al. [47]	Nationwide case–control study	Patients with first-ever stroke from Germany	2009	18–55	762 (37.9)	1247 (62.1)
Putaala et al. [48]	Retrospective	Patients aged 15 to 49 admitted to Helsinki University Central Hospital (Finland)	1008	15–49	380 (37.7)	628 (62.3)
Arntz et al. [49]	Prospective cohort study	Patients recruited in the Follow-Up of Transient Ischemic Attack and Stroke Patients and Unelucidated Risk Factor Evaluation (FUTURE) study and admitted to the Radboud University Nijmegen Medical Centre (Netherlands)	337	18–50	240 (54.6)	153 (45.4)
Ekker et al. [50]	Registry-based cohort study	Cohort was based on two registries: the Dutch nationwide hospital registry and the National Cause of Death Registry (Netherlands)	8444	18–49	4593 (54.4)	3851 (45.6)
Leppert et al. [51]	Retrospective cohort study	Randomly selected sample of a large commercial health insurance database, PharMetrics	5218	15–54	2651 (50.8)	2567 (49.2)
Onwuchekwa et al. [52]	Retrospective, descriptive study	Patients admitted to the University of Port Harcourt Teaching Hospital, Port Harcourt, Rivers State (Nigeria)	54	18–45	26 (48.1)	28 (51.9)
Wu et al. [53]	Retrospective	Patients in a hospital-based population in South Auckland (New Zealand)	131	15–45	66 (50.4)	65 (49.6)
Lasek-Bal et al. [54]	Retrospective	Patients hospitalized in the Department of Neurology and Department of Child Neurology/Division of Pediatrics and Developmental Age Neurology in Katowice (Poland)	141	1–51	67 (47.5)	74 (52.5)
		Total, *n*	21,064/86 *		10,277/45 *	10,843/41 *

* very young group.

## References

[1] Sarecka-Hujar B., Kopyta I. (2018). Poststroke epilepsy: Current perspectives on diagnosis and treatment. Neuropsychiatr. Dis. Treat..

[2] Numis A.L., Fox C.K. (2014). Arterial Ischemic Stroke in Children: Risk Factors and Etiologies. Curr. Neurol. Neurosci. Rep..

[3] George M.G. (2020). Risk Factors for Ischemic Stroke in Younger Adults. Stroke.

[4] Kopyta I., Dobrucka-Głowacka A., Cebula A., Sarecka-Hujar B. (2020). Does the Occurrence of Particular Symptoms and Outcomes of Arterial Ischemic Stroke Depend on Sex in Pediatric Patients? A Pilot Study. Brain Sci..

[5] Sultan S., Schupf N., Dowling M., DeVeber G., Kirton A., Elkind M.S. (2014). Predictors of Cholesterol and Lipoprotein(a) Testing in Children with Arterial Ischemic Stroke. J. Stroke Cerebrovasc. Dis..

[6] Herak D.C., Krleza J.L., Antolic M.R., Horvat I., Djuranovic V., Topic R.Z., Zadro R. (2017). Association of Polymorphisms in Coagulation Factor Genes and Enzymes of Homocysteine Metabolism with Arterial Ischemic Stroke in Children. Clin. Appl. Thromb..

[7] Lopez-Espejo M., Hernandez-Chavez M., Huete I. (2019). Risk factors for in-hospital and follow-up mortality after childhood arterial ischemic stroke. J. Neurol..

[8] Roy-O’Reilley R., McCullough L.D. (2018). Age and Sex Are Critical Factors in Ischemic Stroke Pathology. Endocrinology.

[9] Abanoz Y., Abanoz Y.G., Gündüz A., Uludüz D., Ince B., Yavuz B., Göksan B. (2017). Migraine as a risk factor for young patients with ischemic stroke: A case–control study. Neurol. Sci..

[10] Felling R.J., Sun L.R., Maxwell E.C., Goldenberg N., Bernard T. (2017). Pediatric arterial ischemic stroke: Epidemiology, risk factors, and management. Blood Cells Mol. Dis..

[11] Golomb M.R., Fullerton H.J., Nowak-Gottl U., DeVeber G. (2009). Male Predominance in Childhood Ischemic Stroke. Stroke.

[12] Fullerton H.J., Wu Y.W., Zhao S., Johnston S.C. (2003). Risk of stroke in children: Ethnic and gender disparities. Neurology.

[13] Agrawal N., Johnston S.C., Wu Y.W., Sidney S., Fullerton H.J. (2009). Imaging Data Reveal a Higher Pediatric Stroke Inci-dence Than Prior US Estimates. Stroke.

[14] Mallick A.A., Ganesan V., Kirkham F.J., Fallon P., Hedderly T., McShane T., Parker A.P., Wassmer E., Wraige E., Amin S. (2014). Childhood arterial ischaemic stroke incidence, presenting features, and risk factors: A prospective pop-ulation-based study. Lancet Neurol..

[15] National Center for Injury Prevention and Control, CDC (2018). 10 Leading Causes of Death by Age-Group. https://www.cdc.gov/injury/wisqars/pdf/leading_causes_of_death_by_age_group_2018-508.pdf.

[16] Krishnamurthi R.V., DeVeber G., Feigin V.L., Barker-Collo S., Fullerton H.J., Mackay M.T., O’Callahan F., Lindsay M.P., Kolk A., Lo W. (2015). Stroke Prevalence, Mortality and Disability-Adjusted Life Years in Children and Youth Aged 0-19 Years: Data from the Global and Regional Burden of Stroke 2013. Neuroepidemiology.

[17] Deveber G.A., Kirton A., Booth F.A., Yager J.Y., Wirrell E.C., Wood E., Shevell M., Surmava A.-M., McCusker P., Massicotte M.P. (2017). Epidemiology and Outcomes of Arterial Ischemic Stroke in Children: The Canadian Pediatric Ischemic Stroke Registry. Pediatr. Neurol..

[18] State of New Jersey, Department of Labor & Workforce Development Population Estimates by Single-Year of Age and Sex: April 2010 to July 1, 2019. https://www.nj.gov/labor/lpa/dmograph/est/est_index.html.

[19] Naeye R.L., Burt L.S., Wright D.L., A Blanc W., Tatter D. (1971). Neonatal mortality, the male disadvantage. Pediatry.

[20] Aubenque M. (1989). Indice de masculinite a la naissance: Apercu retrospectif et commentaires [The sex ratio at birth: A ret-rospective review and commentary]. J. Soc. Stat. Paris..

[21] Bohmer M., Niederstadt T., Heindel W., Wildgruber M., Sträter R., Hanning U., Kemmling A., Sporns P.B. (2019). Impact of arterial ischemic stroke standardized classification and diagnostic evaluation classification on further course of ar-teriopathy and recurrence of childhood stroke. Stroke.

[22] Steinlin M., Bigi S., Stojanovski B., Gajera J., Regényi M., El-Koussy M., Mackay M.T. (2017). Swiss NeuroPediatric Stroke Registry. Focal Cerebral Arteriopathy: Do Steroids Improve Outcome?. Stroke.

[23] Per H., Unal E., Poyrazoğlu H.G., Ozdemir M.A., Donmez H., Gümüş H., Üzüm K., Canpolat M., Akyildiz B.N., Coşkun A. (2014). Childhood Stroke: Results of 130 Children from a Reference Center in Central Anatolia, Turkey. Pediatr. Neurol..

[24] Rambaud T., Legris N., Bejot Y., Bellesme C., Lapergue B., Jouvent E., Pico F., Smadja D., Zuber M., Crozier S. (2020). Acute ischemic stroke in adolescents. Neurol..

[25] Nowak-Göttl U., Sträter R., Kosch A., Von Eckardstein A., Schobess R., Luigs P., Nabel P., Vielhaber H., Kurnik K., Junker R. (2001). The plasminogen activator inhibitor (PAI)-1 promoter 4G/4G genotype is not associated with ischemic stroke in a population of German children. Eur. J. Haematol..

[26] Masri A., Al-Ammouri I. (2016). Clinical presentation, etiology, and outcome of stroke in children: A hospital-based study. Brain Dev..

[27] Gelfand A.A., Croen L.A., Torres A.R., Wu Y.W. (2013). Genetic Risk Factors for Perinatal Arterial Ischemic Stroke. Pediatr. Neurol..

[28] Golomb M.R., Dick P.T., MacGregor D.L., Curtis R., Sofronas M., DeVeber G.A. (2004). Neonatal arterial ischemic stroke and cerebral sinovenous thrombosis are more commonly diagnosed in boys. J. Child Neurol..

[29] Salih M.A., Abdel-Gader A.G., Al-Jarallah A.A., Kentab A.Y., Alorainy I.A., Hassan H.H., Al-Nasser M.N. (2006). Perinatal stroke in Saudi children. Clinical features and risk factors. Saudi Med. J..

[30] Sträter R., Becker S., von Eckardstein A., Heinecke A., Gutsche S., Junker R., Kurnik K., Schobess R., Nowak-Göttl U. (2002). Prospective assessment of risk factors for recurrent stroke during childhood—A 5-year follow-up study. Lancet.

[31] Sebire G., Tabarki B., Saunders D.E., Leroy I., Liesner R., Saint-Martin C., Husson B., Williams A.N., Wade A., Kirkham F.J. (2005). Cerebral venous sinus thrombosis in children: Risk factors, presentation, diagnosis and outcome. Brain.

[32] Kenet G., Sadetzki S., Murad H., Martinowitz U., Rosenberg N., Gitel S., Rechavi G., Inbal A. (2000). Factor V Leiden and Antiphospholipid Antibodies Are Significant Risk Factors for Ischemic Stroke in Children. Stroke.

[33] Kenet G., Kirkham F., Niederstadt T., Heinecke A., Saunders D., Stoll M., Brenner B., Bidlingmaier C., Heller C., Knöfler R. (2007). Risk factors for recurrent venous thromboembolism in the European collaborative paediatric database on cerebral venous thrombosis: A multicentre cohort study. Lancet Neurol..

[34] Bonduel M., Sciuccati G., Hepner M., Pieroni G., Torres A.F., Frontroth J.P., Tenembaum S. (2006). Arterial Ischemic Stroke and Cerebral Venous Thrombosis in Children: A 12-Year Argentinean Registry. Acta Haematol..

[35] Ozyurek E., Balta G., Degerliyurt A., Parlak H., Aysun S., Gürgey A. (2007). Significance of Factor V, Prothrombin, MTHFR, and PAI-1 Genotypes in Childhood Cerebral Thrombosis. Clin. Appl. Thromb..

[36] Hills N.K., Johnston S.C., Sidney S., Zielinski B.A., Fullerton H.J. (2012). Recent trauma and acute infection as risk factors for childhood arterial ischemic stroke. Ann. Neurol..

[37] Helmuth I.G., Mølbak K., Uldall P.V., Poulsen A. (2018). Post-varicella Arterial Ischemic Stroke in Denmark 2010 to 2016. Pediatr. Neurol..

[38] Fullerton H.J., Luna J.M., Wintermark M., Hills N.K., Tokarz R., Li Y., Glaser C., DeVeber G.A., Lipkin W.I., Elkind M.S.V. (2017). Parvovirus B19 Infection in Children with Arterial Ischemic Stroke. Stroke.

[39] Amlie-Lefond C., Bernard T.J., Sébire G., Friedman N.R., Heyer G.L., Lerner N.B., de Veber G., Fullerton H.J. (2009). Pre-dictors of Cerebral Arteriopathy in Children with Arterial Ischemic Stroke: Results of the International Pediatric Stroke Study. Circulation.

[40] Szaflarski J., Allendorfer J., Byars A., Vannest J., Dietz A., Hernando K., Holland S. (2014). Age at stroke determines post-stroke language lateralization. Restor. Neurol. Neurosci..

[41] Kissela B.M., Khoury J.C., Alwell K., Moomaw C.J., Woo D., Adeoye O., Flaherty M.L., Khatri P., Ferioli S., Rosa F.D.L.R.L. (2012). Age at stroke: Temporal trends in stroke incidence in a large, biracial population. Neurology.

[42] Bergman E.-M., Henriksson K.M., Åsberg S., Farahmand B., Terént A. (2015). National registry-based case-control study: Comorbidity and stroke in young adults. Acta Neurol. Scand..

[43] Martínez-Sánchez P., Fuentes B., Fernández-Domínguez J., Ortega-Casarrubios M.D.L. (2011). Ángeles; Aguilar-Amar, M.J.; Abenza-Abildúa, M.J.; Idrovo-Freire, L.; Diez-Tejedor, E.; Gimeno, B.E.F. Young Women Have Poorer Outcomes than Men after Stroke. Cerebrovasc. Dis..

[44] Spengos K., Vemmos K. (2010). Risk factors, etiology, and outcome of first-ever ischemic stroke in young adults aged 15 to 45—The Athens young stroke registry. Eur. J. Neurol..

[45] Kwon S.U., Kim J.S., Lee J.H., Lee M.C. (2000). Ischemic stroke in Korean young adults. Acta Neurol. Scand..

[46] Tang M., Yao M., Zhu Y., Peng B., Zhou L., Ni J. (2020). Sex differences of ischemic stroke in young adults—A single-center Chinese cohort study. J. Stroke Cerebrovasc. Dis..

[47] Aigner A., Grittner U., Rolfs A., Norrving B., Siegerink B., Busch M.A. (2017). Contribution of Established Stroke Risk Factors to the Burden of Stroke in Young Adults. Stroke.

[48] Putaala J., Metso A.J., Metso T.M., Konkola N., Kraemer Y., Haapaniemi E., Kaste M., Tatlisumak T. (2009). Analysis of 1008 Consecutive Patients Aged 15 to 49 With First-Ever Ischemic Stroke. Stroke.

[49] Arntz R.M., Broek S.M.V.D., Van Uden I.W., Ghafoorian M., Platel B., Rutten-Jacobs L.C., Maaijwee N.A., Schaapsmeerders P., Schoonderwaldt H.C., Van Dijk E.J. (2016). Accelerated development of cerebral small vessel disease in young stroke patients. Neurology.

[50] Ekker M.S., Verhoeven J.I., Vaartjes I., Jolink W.M.T., Klijn C.J.M., De Leeuw F.-E. (2019). Association of Stroke Among Adults Aged 18 to 49 Years with Long-term Mortality. JAMA.

[51] Leppert M.H., Ho P.M., Burke J., Madsen T.E., Kleindorfer D., Sillau S., Daugherty S., Bradley C.J., Poisson S.N. (2020). Young Women Had More Strokes Than Young Men in a Large, United States Claims Sample. Stroke.

[52] Onwuchekwa A., Asekomeh E., Onwuchekwa R. (2009). Stroke in young Nigerian adults. J. Vasc. Nurs..

[53] Wu T.Y., Kumar A., Wong E.H. (2012). Young ischaemic stroke in South Auckland: A hospital-based study. New Zealand Med J..

[54] Lasek-Bal A., Kopyta I., Warsz-Wianecka A., Puz P., Łabuz-Roszak B., Zaręba K. (2018). Risk factor profile in patients with stroke at a young age. Neurol. Res..

[55] Tuckuviene R., Christensen A., Helgestad J., Johnsen S., Kristensen S. (2010). Paediatric arterial ischaemic stroke and cerebral sinovenous thrombosis in Denmark 1994–2006: A nationwide population-based study. Acta Paediatr..

[56] Carey S., Wrogemann J., Booth F.A., Rafay M.F. (2017). Epidemiology, Clinical Presentation, and Prognosis of Posterior Circulation Ischemic Stroke in Children. Pediatr. Neurol..

[57] Colsch R., Lindseth G. (2018). Unique Stroke Symptoms in Women: A Review. J. Neurosci. Nurs..

[58] Labiche L.A., Chan W., Saldin K.R., Morgenstern L.B. (2002). Sex and acute stroke presentation. Ann. Emerg. Med..

[59] Khan F., Ibrahim A. (2018). Gender differences in risk factors, clinical presentation, and outcome of stroke: A secondary analysis of previous hospital-based study in Qatar. Libyan J. Med Sci..

[60] Von Sarnowski B., Schminke U., Grittner U., Tanislav C., Böttcher T., Hennerici M.G., Tatlisumak T., Putaala J., Kaps M., Fazekas F. (2017). Posterior versus Anterior Circulation Stroke in Young Adults: A Comparative Study of Stroke Aetiologies and Risk Factors in Stroke among Young Fabry Patients (sifap1). Cerebrovasc. Dis..

[61] Kuruvilla A., Bhattacharya P., Rajamani K., Chaturvedi S. (2011). Factors Associated With Misdiagnosis of Acute Stroke in Young Adults. J. Stroke Cerebrovasc. Dis..

[62] Breitweg I., Von Stülpnagel C., Pieper T., Lidzba K., Holthausen H., Staudt M., Kluger G. (2017). Early seizures predict the development of epilepsy in children and adolescents with stroke. Eur. J. Paediatr. Neurol..

[63] Lee E.H., Yum M.-S., Ko T.-S. (2011). Risk Factors and Clinical Outcomes of Childhood Ischemic Stroke in a Single Korean Tertiary Care Center. J. Child Neurol..

[64] Roivainen R., Haapaniemi E., Putaala J., Kaste M., Tatlisumak T. (2013). Young adult ischaemic stroke related acute symptomatic and late seizures: Risk factors. Eur. J. Neurol..

[65] Chen T.-C., Chen Y.-Y., Cheng P.-Y., Lai C.-H. (2012). The incidence rate of post-stroke epilepsy: A 5-year follow-up study in Taiwan. Epilepsy Res..

[66] De Schryver E.L., Kappelle L.J., Jennekens-Schinkel A., Boudewyn Peters A.C. (2000). Prognosis of ischemic stroke in childhood: A long-term follow-up study. Dev. Med. Child. Neurol..

[67] Morais N.M.M., Ranzan J., Riesgo R.S. (2012). Predictors of Epilepsy in Children with Cerebrovascular Disease. J. Child Neurol..

[68] Kopyta I., Sarecka-Hujar B., Skrzypek M. (2015). Post-stroke epilepsy in Polish paediatric patients. Dev. Med. Child Neurol..

[69] Westmacott R., MacGregor D., Askalan R., DeVeber G. (2009). Late Emergence of Cognitive Deficits After Unilateral Neonatal Stroke. Stroke.

[70] Öztürk Ş., Ege F., Ekmekci H. (2014). Language Disorders due to Posterior System Strokes—An Ignored Dysfunction. Arch. Neuropsychiatry.

[71] Fullerton H.J., Wintermark M., Hills N.K., Dowling M.M., Tan M., Rafay M.F., Elkind M.S.V., Barkovich A.J., DeVeber G.A., Plumb P.A. (2016). Risk of Recurrent Arterial Ischemic Stroke in Childhood. Stroke.

[72] Uohara M.Y., Beslow L.A., Billinghurst L., Jones B.M., Kessler S.K., Licht D.J., Ichord R.N. (2017). Incidence of Recurrence in Posterior Circulation Childhood Arterial Ischemic Stroke. JAMA Neurol..

[73] Rafay M.F., Shapiro K.A., Surmava A.-M., DeVeber G.A., Kirton A., Fullerton H.J., Amlie-Lefond C., Weschke B., Dlamini N., Carpenter J.L. (2020). Spectrum of cerebral arteriopathies in children with arterial ischemic stroke. Neurol..

[74] Geng C., Lin Y., Tang Q., Tang Y., Wang X., Zhou J.-S., Yang J., Zheng D., Zhang Y.-D. (2018). Sex differences in clinical characteristics and 1-year outcomes of young ischemic stroke patients in East China. Ther. Clin. Risk Manag..

[75] Rutten-Jacobs L.C.A., Arntz R.M., Maaijwee N.A.M., Schoonderwaldt H.C., Dorresteijn L.D., Van Dijk E.J., De Leeuw F.-E. (2013). Long-term Mortality After Stroke Among Adults Aged 18 to 50 Years. JAMA.

[76] Lutski M., Zucker I., Shohat T., Tanne D. (2017). Characteristics and Outcomes of Young Patients with First-Ever Ischemic Stroke Compared to Older Patients: The National Acute Stroke ISraeli Registry. Front. Neurol..

[77] Arntz R., Rutten-Jacobs L., Maaijwee N., Schoonderwaldt H., Dorresteijn L., Van Dijk E., De Leeuw F.-E. (2013). Post-Stroke Epilepsy in Young Adults: A Long-Term Follow-Up Study. PLoS ONE.

[78] Naess H., Nyland H.I., Thomassen L., Aarseth J., Myhr K.-M. (2004). Long-term outcome of cerebral infarction in young adults. Acta Neurol. Scand..

[79] Naess H., Nyland H.I., Thomassen L., Aarseth J., Myhr K.-M. (2005). Mild depression in young adults with cerebral infarction at long-term follow-up: A population-based study. Eur. J. Neurol..

[80] Lim T.S., Lee J.S., Yoon J.H., Moon S.Y., Joo I.S., Huh K., Hong J.M. (2017). Cigarette smoking is an independent risk factor for post-stroke delirium. BMC Neurol..

[81] Simpkins J.W., Rajakumar G., Zhang Y.-Q., Simpkins C.E., Greenwald D., Yu C.J., Bodor N., Day A.L. (1997). Estrogens may reduce mortality and ischemic damage caused by middle cerebral artery occlusion in the female rat. J. Neurosurg..

[82] Castelló-Ruiz M., Torregrosa G., Burguete M.C., Miranda F.J., Centeno J.M., López-Morales M.A., Gasull T., Alborch E. (2014). The selective estrogen receptor modulator, bazedoxifene, reduces ischemic brain damage in male rat. Neurosci. Lett..

[83] Manwani B., McCullough L.D. (2011). Sexual Dimorphism in Ischemic Stroke: Lessons from the Laboratory. Women’s Health.

[84] Ritzel R.M., Capozzi L.A., McCullough L.D. (2013). Sex, stroke, and inflammation: The potential for estrogen-mediated immunoprotection in stroke. Horm. Behav..

[85] Liu F., McCullough L.D. (2012). Interactions between age, sex, and hormones in experimental ischemic stroke. Neurochem. Int..

[86] Ahnstedt H., McCullough L.D., Cipolla M.J. (2016). The Importance of Considering Sex Differences in Translational Stroke Research. Transl. Stroke Res..

[87] Normann S., De Veber G., Fobker M., Langer C., Kenet G., Bernard T.J., Fiedler B., Sträter R., Goldenberg N.A., Nowak-Göttl U. (2009). Role of endogenous testosterone concentration in pediatric stroke. Ann. Neurol..

[88] Zak I., Sarecka-Hujar B., Kopyta I., Emich-Widera E., Marszal E., Wendorff J., Jachowicz-Jeszka J. (2009). The T Allele of the 677C>T Polymorphism ofMethylenetetrahydrofolate ReductaseGene is Associated with an Increased Risk of Ischemic Stroke in Polish Children. J. Child Neurol..

[89] Coen Herak D., Čeri A., Grzunov A., Krleza J.L., Radic Antolic M., Horvat I., Đuranović V., Barisic N., Zrinski Topić R., Zadro R. Gender specific effect of the fxiii-a val34leu polymorphism on the risk for childhood and perinatal arterial ischemic stroke. Proceedings of the 2nd International Factor XIII Workshop.

[90] Sarecka-Hujar B., Zak I., Emich-Widera E., Kopyta I., Pilarska E., Pienczk-Reclawowicz K. (2010). Association analysis of the E-selectin 98G > T polymorphism and the risk of childhood ischemic stroke. Cell Biochem. Funct..

[91] Gromadzka G., Baranska-Gieruszczak M., Sarzynska-Dlugosz I., Ciesielska A., Czlonkowska A. (2007). The APOE pol-ymorphism and 1-year outcome in ischemic stroke: Genotype-gender interaction. Acta Neurol. Scand..

[92] Martiskainen M., Oksala N., Pohjasvaara T., Kaste M., Oksala A., Karhunen P.J., Erkinjuntti T. (2014). Βeta-fibrinogen gene promoter A −455 allele associated with poor longterm survival among 55–71 years old Caucasian women in Finnish stroke cohort. BMC Neurol..

[93] Joly B., Menard A.-L., Ozkul-Wermester O., Triquenot-Bagan A., Guegan-Massardier E., Borg J.-Y., Le Cam-Duchez V. (2014). Involvement of Arg306 mutation in factor V gene in two young men with ischemic stroke. Blood Coagul. Fibrinolysis.

